# Perceived needs and health-related quality of life in people with schizophrenia and metabolic syndrome: a “real-world” study

**DOI:** 10.1186/s12888-016-1005-4

**Published:** 2016-11-21

**Authors:** Leticia Medeiros-Ferreira, José Blas Navarro-Pastor, Antonio Zúñiga-Lagares, Rosanna Romaní, Elisenda Muray, Jordi E. Obiols

**Affiliations:** 1 Department of Clinical and Health Psychology, Faculty of Psychology, Universitat Autònoma de Barcelona/Nou Barris Nord Mental Health Center, Paseo Valldaura 214- bajos, 08042 Barcelona, Spain; 2 Nou Barris Nord Mental Health Center, Paseo Valldaura 214- bajos, 08042 Barcelona, Spain; 3 Department of Psychobiology and Methodology of Health Sciences, Faculty of Psychology, Universitat Autònoma de Barcelona, Barcelona, Spain

**Keywords:** Schizophrenia, Metabolic syndrome, Health-related quality of life, Perceived needs

## Abstract

**Background:**

The complexity of schizophrenia lies in the combination of psychiatric, somatic and social needs requiring care. The aim of the study was to compare perceived needs between groups with absence/presence of metabolic syndrome (MetS) and to analyze the relationship between needs, health-related quality of life (HRQoL) and MetS in people with schizophrenia or schizoaffective disorder.

**Methods:**

A “real-world” cross-sectional study was set up with a comprehensive framework including the following, needs for care (Camberwell Assessment of Need Interview [CAN]), HRQoL (Euro Qol-5D Questionnaire), sociodemographic data, lifestyle habits, psychopathology (Positive And Negative Syndrome Scale [PANSS]), global functioning (Global Assessment of Functioning Scale [GAF]), anthropometric measurements and blood test results were assessed for an outpatient sample (*n* = 60).

**Results:**

The mean number of needs (given by CAN) was identified for both groups. Patients with MetS rated a higher number of needs compared to the group without this condition. Mobility problems (given by EQ-5D) were negatively associated with the number of total and unmet needs. For participants with MetS, HRQoL was related to the number of needs and unmet needs. For people with MetS, positive symptomatology score (given by PANSS) was related to the number of needs and met needs and general symptomatology was associated with total, met and unmet needs. For individuals without MetS, the global functioning score (given by GAF) was significantly inversely related with total, met and unmet needs.

**Conclusions:**

Needs and HRQoL, as well as general symptomatology, were related only in patients with MetS. This has implications for treatment planning at the individual and organizational levels. An analysis of both physical and mental needs could provide a starting point for the extension of facilities in the health care system in order to reach the goal of improving quality of life.

## Background

The complexity of schizophrenia lies in the combination of psychiatric, somatic and social needs requiring care [1, 2]. “Needs for care” has been summarized as “the requirements of individuals to enable them to achieve, maintain or restore an acceptable level of social independence or quality of life (QoL)” [3]. A need is met when an intervention is efficacious and the patient is offered effective help; a need is unmet when an intervention has no, or little, effect [4]. The assessment of needs from a health care service perspective can be beneficial in the planning and provision of individual care [5]. In accordance with this idea, Ochoa et al. (on behalf of the NEDES Group) [6] evaluated outpatients with schizophrenia using the Camberwell Assessment of Need (CAN). The most prevalent domains were related to psychotic symptoms, household skills, need for help with food and with information about their condition and treatment. Zúñiga et al. [7] examined needs in a sample of outpatients suffering from psychotic and other disorders and found similar results to those described by Ochoa et al. [6] and others [8–13]. Factors associated with a higher number of needs were low socioeconomic class, older age, poorer global functioning and the presence of psychotic disorder.

Schizophrenia produces a range of disabilities in everyday life and unmet needs are associated with poor QoL [6, 13–16]. QoL, a multidimensional construct that includes subjective well-being and objective mental and physical function indicators, has been recognized as an important outcome of schizophrenia treatment [17, 18]. Health-related quality of life (HRQoL) is restricted to patients’ self-perception of symptomatology, disability and functional status related to physical and/or mental health [19].

In this context, the Clinical Antipsychotic Trials of Intervention Effectiveness Schizophrenia Trial (CATIE) [20] showed a correlation between schizophrenia, QoL and the metabolic syndrome (MetS). MetS comprises a spectrum of medical disorders including abdominal obesity, insulin resistance, dyslipidemia and elevated blood pressure and has been associated with an increased risk of developing type 2 diabetes mellitus and cardiovascular disease. The CATIE study confirmed the relationship between physical comorbidity and self-perception of poor physical health, as did the study of Dixon et al. [21]. A higher functioning score as measured by the Global Assessment of Functioning (GAF) scale correlated with a better subjective rating of QoL in a longitudinal outcome study [22]. Medeiros-Ferreira et al. [23] studied the relationship between MetS, HRQoL and global functioning in schizophrenia and schizoaffective disorder. The prevalence of MetS was 36.8% and was correlated with higher body mass index (BMI), older age, unemployment status and better self-care (probably due to increased awareness of self-image). Patients with MetS who engaged in physical activity reported a subjective perception of better health and, consequently, better HRQoL. People with schizophrenia have poorer access to and quality of health care [24, 25]. It has been shown that 50 % of individuals with schizophrenia exhibit at least one physical or other psychiatric comorbidity [26–28]. Many studies have confirmed the high prevalence of MetS in schizophrenia in different contexts, but with very disparate results (11-69 %), depending on the sample and the methods used [29]. It is well known that the combination of biological risk, lifestyle factors and treatment favors the development of MetS in such patients [23, 28, 30–32]. The importance of monitoring MetS in people with schizophrenia in order to reduce the associated risks has been reiterated by consensus panels throughout the world [31, 33].

While needs can be seen as the starting point for developing interventions for people with schizophrenia, the ultimate goal is to improve QoL. The aim of the present study was therefore to compare needs between the groups with absence/presence of MetS and to analyze the relationship between perceived needs for care, HRQoL, lifestyle habits, psychopathology, global functioning and MetS in patients with schizophrenia or schizoaffective disorder in a psychiatric outpatient sample. We hypothesized that individuals diagnosed with schizophrenia or schizoaffective disorder and also with MetS have a higher number of needs and more unmet needs; poorer HRQoL, lifestyle habits and global functioning and more severe psychopathology than those without this condition.

## Methods

### Study design and procedure

This was a “real-world” cross-sectional study. A “real-world” study is one that is non-interventional with an unrestricted population, where the basis is the clinical practice and the focus is the need for changes in practice. The sample was composed of 60 consecutive patients (23 with MetS and 37 without) attending the Severe Mental Disorder Program and, more specifically, the Metabolic Syndrome Protocol at a public mental health center in Barcelona, Spain. The inclusion criteria were the following: outpatients who had received antipsychotic medication for at least 12 weeks, had had a blood test in the previous 3 months and had given informed consent. The exclusion criterion was incapacity to understand the questions formulated in Spanish. Those individuals not included in analysis showed no differences with respect to the sample subjects in terms of sociodemographic and clinical characteristics.

### Data collection

The interviews were conducted by the patients’ own psychiatrist (LMF) and reference nurse (RR) in routine individual sessions (30-45 min) at the mental health center. One clinician-investigator (AZL) confirmed the diagnosis of schizophrenia or schizoaffective disorder using the Structured Clinical Interview for DSM-IV Axis I Disorders (SCID-I) [34] and the other clinician-investigator (LMF) administered the protocol, ordered the blood tests and confirmed the diagnosis of MetS using the modified National Cholesterol Education Program Adult Treatment Panel Criteria (NCEP-ATP-III criteria) [35]. The nursing staff (mainly RR) collected all anthropometric measurements and the perceived needs (given by CAN) were identified by the social worker (EM). About 2 months were necessary to collect all information due to the routine assessment format. The investigators were totally familiar with the use of all the chosen instruments. The study complied with the principles contained in the Declaration of Helsinki [36] and was reviewed and approved by the institutional ethics board at the study center.

### Measurement instruments

All patients were evaluated using the following instruments:

A sociodemographic and clinical questionnaire that included information on psychiatric history, comorbidity, anthropometric measurements (weight, height, BMI, waist circumference and blood pressure), metabolic profile (cholesterol, triglycerides and fasting glucose) and lifestyle (diet, physical exercise, substance use) and antipsychotic treatment.

The Positive and Negative Syndrome Scale (PANSS), Spanish version [37, 38], divided into positive (PANSS-P), negative (PANSS-N) and general (PANSS-G) symptomatology. The psychometric properties of the PANSS are currently well documented showing good validity and reliability with good internal consistency of its five-factor structure (Cronbach’s alpha >0.70) [37].

The Global Assessment of Functioning (GAF) Scale [39]. Studies of concurrent validity of the GAF with schizophrenia samples indicate that the GAF is a valid measure of global psychological, social, and occupational functioning [40].

The EuroQol-5 Dimensions (EQ-5D), Spanish version [41, 42], divided into 2 sections: 1) a descriptive system that assesses HRQoL in 5 dimensions (mobility, self-care, usual activities, pain/discomfort, and anxiety/depression), each with 3 levels of severity (value 1 = no problems, value 2 = some problems, value 3 = severe problems); 2) a visual analogue scale (VAS) in the form of a “thermometer” (value 0 = worst and value 100 = best imaginable health status). It describes the self-perception of health on each dimension on the day of administration. In our sample, reliability for the 5 dimensions evaluated was low (Cronbach’s alpha = 0.664), although it is mainly due to the reduced number of items not to inconsistencies of the answers.

The CAN Interview, Spanish version [1, 9, 43], which evaluates the presence of needs for care in 22 domains in the previous month. The number of total, met and unmet needs can be calculated for each domain and recorded from different perspectives (service user and/or staff or caregiver). We considered the patient’s perspective only. In our sample, Cronbach’s alpha = 0.801 was obtained.

### Data analysis

Statistical analyses were performed using the SPSS 20.0 software package (SPSS Inc., Chicago, Illinois, USA) and the significance level used was *p* < 0.05 (2-tailed). The EQ-5D dimensions were dichotomized into absent/present (value 1 = absence and value 2 + 3 = presence of problem) and the number of problems was added up under the label “EQ-5D Total Score”. The frequency distributions of met and unmet perceived needs were determined and the mean number of total, met and unmet needs was calculated and compared between the groups with absence/presence of MetS. First, data analyses consisting of the descriptive statistics of all the measurements were done. Subsequently, the Student’s *t*-test was used to compare means between continuous variables, the chi-squared test to study the association between nominal variables and the Mantel-Haenszel test for ordinal variables. Pearson correlations were calculated to measure the degree of association between total, met, unmet needs and quality of live, psychotic symptomatology. Given the absence of statistical and clinical differences between patients with and without MetS (Table 1), except for the physical measurements which defines MetS (dyslipidemia, diabetes mellitus), there was no necessity to statistically adjust for any confounding variables.

**Table 1:** Sociodemographic and clinical variables according to the absence (*N* = 37)/presence (*N* = 23) of metabolic syndrome

MetS	No	Yes	t/Chi^2(A)^	*p*
Age (mean, SD)	41.7 (10.9)	46.0 (10.1)	1.54	.130
Sex (*N* = 60)			.582	.445
Male	62.2 %	52.2 %		
Female	37.8 %	47.8 %		
Employment Status (*N* = 60)			.353	.553
Active	18.9 %	13.0 %		
Unemployed	81.1 %	87.0 %		
Living Arrangements			.023	.879
Living with parents	86.5 %	91.3 %		
Others	13.5 %	8.7 %		
Education (*N* = 60)			.244	.622
Primary school	37.8 %	43.5 %		
Secondary school	24.3 %	17.4 %		
Medium studies (professional college)	18.9 %	30.4 %		
University	18.9 %	8.7 %		
Years since diagnosis (*N* = 59)			.570	.450
Less than 5 years	19.4 %	8.7 %		
5-10 years	6.0 %	21.7 %		
10-15 years	22.2 %	21.7 %		
More than 15 years	41.7 %	47.8 %		
Lifestyle habits (*N* = 60)				
Smoking	56.8 %	73.9 %	1.79	.180
Alcoholism	24.3 %	26.1 %	.024	.878
Abuse of other substances/drugs^(B)^	29.7 %	17.4 %	1.15	.283
Diet (*N* = 59)				
Unrestricted calories	80.6 %	65.2 %	1.74	.187
Unrestricted salt	66.7 %	56.5 %	.618	.432
Unrestricted fibers	66.7 %	65.2 %	.013	.909
Unrestricted saturated fat	44.4 %	26.1 %	2.02	.155
Physical activity	69.4 %	65.2 %	.115	.735
Medical history (*N* = 60)				
Cardiovascular disease	2.7 %	30.4 %	9.44	.002
Dyslipidemia	8.1 %	30.4 %	5.09	.024
Diabetes mellitus	13.5 %	43.5 %	6.79	.009
Psychopharmacological Treatment (*N* = 60)				
Antipsychotics (AP)- Monotherapy	54.1 %	30.4 %	3.19	.074
Antipsychotics (AP)- Polytherapy	45.9 %	69.6 %		
Antidepressants (AD)	40.5 %	39.1 %	.012	.914
Anxiolytics/Hypnotics (AN)	59.5 %	69.6 %	.624	.430
Mood stabilizers (MS)	32.4 %	43.5 %	.745	.388
Others^(C)^	21.6 %	26.1 %	.158	.691

(A): Student’s t for mean comparison; Chi^2^ for nominal and Mantel-Haenszel for ordinal variables

(B): Caffeine, cannabis, cocaine, hypnotics

(C): Anticholinergics, treatment for alcohol dependence (naltrexone and dissulfiram)

### Results

The sociodemographic and clinical characteristics according to the absence/presence of MetS in participants with schizophrenia or schizoaffective disorder are shown in Table 1. The sample was composed mostly of unemployed men living with their parents, with a primary school level of education, who had been diagnosed more than 15 years earlier, had been treated with more than 2 antipsychotics and had a history of diabetes, cardiovascular disease and dyslipidemia. Most of the participants were smokers and did not restrict calorie or salt intake, but were in the habit of eating fibre and engaging in physical activity. We found that MetS was associated with a history of cardiovascular disease (*p* = 0.00), diabetes (*p* = 0.00) and dyslipidemia (*p* = 0.02).

The percentages of needs in the 22 CAN areas rated by the patient and their relation to MetS are given in Table 2. Patients rated a mean of 5.5 total needs (SD = 3.43) with a score of 4 met (SD = 2.48) and 1.5 unmet needs (SD = 1.83). Although there were no significant differences between absence/presence of MetS in the percentages of needs, we found some high values in some domains. In the group with MetS, there were 18.1% fewer patients with no perceived needs in the physical health domain and 18.7 % fewer patients with met needs in the psychotic symptoms domain. On the other hand, we found 25.6 % more patients with met needs in the company domain in the group without MetS.

**Table 2:** Percentages (%) of subjects with no needs, met needs or unmet needs for 22 CAN domains (user's perspective) and stratified by presence/absence of metabolic syndrome

All sample (*N* = 60)				MetS Yes vs No^c^			
CAN domains	No needs (%)	Met needs (%)	Unmet needs (%)	No needs (%)	Met needs (%)	Unmet needs (%)	*P*
1. Accommodation	95.0	3.3	1.7	-6.0	8.7	-2.7	.691
2. Food	56.7	33.3	10.0	-0.3	2.4	-2.1	.917
3. Looking after the home	45.8	44.1	10.2	-15	2.3	12.8	.120
4. Self-care	86.7	11.7	1.7	0.5	-4.8	4.3	.718
5. Daytime activities^a^	52.5	25.4	22.0	13.7	-13.2	-0.5	.516
6. Physical health	63.3	30.0	6.7	-18.1	7.8	10.3	.084
7. Psychotic symptoms	41.7	55.0	3.3	17.1	-18.7	1.6	.296
8. Information^a^	85.0	11.7	1.7	-10.9	9.3	4.3	.844
9. Psychological distress	26.7	41.7	31.7	-8.0	-4.1	12.1	.325
10. Safety to self	85.0	11.7	3.3	3.2	2.2	-5.4	.491
11. Safety to others	88.3	8.3	3.3	4.8	-6.5	1.6	.788
12. Alcohol	95.0	5.0	0.0	1.1	-1.1	0.0	.856
13. Drugs	93.3	5.0	1.7	3.8	-1.1	-2.7	.466
14. Company^b^	50.0	23.3	25.0	-17.7	25.6	-12.3	.265
15. Intimate relationships^b^	73.3	21.7	3.3	-13.2	7.2	1.6	.132
16. Sexual expression^b^	71.7	15.0	10.0	-10.5	3.9	4.9	.526
17. Child care	96.6	3.4	0.0	5.4	-5.4	0.0	.271
18. Basic education	91.7	6.7	1.7	-0.6	3.3	-2.7	.822
19. Telephone	90.0	8.3	1.7	-4.9	7.6	-2.7	.821
20. Transport	85.0	10.0	5.0	-3.9	-2.1	6.0	.470
21. Money	68.3	23.3	8.3	-19.2	18.6	0.6	.248
22. Welfare benefits	98.3	1.7	0.0	-4.3	4.3	0.0	.205

^a^ *N* = 59

^b^Unknown need status was given for each domain due to the user’s refusal to discuss the domain and it was statistically treated as No need (for further details, please consult www.researchintorecovery.com)

^c^Indicates the difference for each possible answer (no need, met and unmet need) between patients with and without MetS. For example, for “6.Physical health”, the value -18.1% indicates that there are18.1% fewer of patients with no needs in the group with MetS than in the group without MetS

As can be seen, in Table 3, there was no association between the number of needs and the presence of MetS. The mean number of total needs of the participants with MetS was 5.95 (SD = 3.97), with a score of 4.26 met (SD = 2.73) and 1.69 unmet needs (SD = 2.20).

**Table 3:** Comparison of total number of needs, met and unmet needs according to the absence/presence of metabolic syndrome

Mean difference						
CAN score	MetS	N	Mean of perceived needs	SD	*p*	95 % CI
Total Needs	No	37	5.21	3.12	.425	-2.58 to 1.10
	Yes	23	5.95	3.97		
Met Needs	No	37	3.81	2.36	.503	-1.78 to .885
	Yes	23	4.26	2.73		
Unmet Needs	No	37	1.40	1.58	.556	-1.27 to .691
	Yes	23	1.69	2.20		

The associations between the number of total, met and unmet needs according to the presence/absence of MetS and age, EQ-5D total score, EQ-VAS, GAF and PANSS scores are shown in Table 4. For participants with MetS, age was associated with the number of needs (*p* = 0.463) and unmet needs (*p* = 0.467). The EQ-5D total score showed no significant association with MetS. However, mobility problems (EQ-5D dimension) were negatively associated with the number of needs (*p* = 0.01) and unmet (*p* = 0.03) needs. For people with MetS, HRQoL was related to the number of needs (*p* = 0.028) and unmet (*p* = 0.043) needs. For patients with MetS, the PANSS-P score was related to the number of needs (*p* = 0.018) and met needs (*p* = 0.020) and PANSS-G was associated with the number of needs (*p* = 0.000), met needs (*p* = 0.022) and unmet needs (*p* = 0.001). The PANSS-N score was not associated with the number of needs in participants with MetS. For individuals without MetS, the GAF score was significantly inversely related to the number of total (*p* = 0.005), met (*p* = 0.039) and unmet needs (*p* = 0.014).

**Table 4:** Correlations between the perceived needs and age, health-related quality of life(A), global functioning(B) and symptomatology(C), stratified by absence/presence of metabolic syndrome

CAN score	MetS	N	AGE	EQ-5D total score	EQ-VAS	GAF	PANSS-P	PANSS-N	PANSS-G
Total Needs	No	36	.103	.118	-.200	-.454*	.052	.261	.220
	Yes	23	.463*	.273	-.457*	-.260	.490*	.220	.680*
Met Needs	No	36	.190	.093	-.103	-.346*	.069	.172	.092
	Yes	23	.296	.292	-.320	-.299	.483*	.055	.475*
Unmet Needs	No	36	-.080	.102	-.246	-.406*	.007	.270	.299*
	Yes	23	.467*	.129	-.426*	-.185	.283	.328	.636*

(A): HRQoL- EQ-5D + EQ-VAS. (B): GAF. (C): PANSS-P + PANSS-N + PANSS-G. *: *p* < 0.05

### Discussion

MetS is an important topic for research and clinical practice in the field of schizophrenia. The same is true of the study of QoL and needs assessment, both of which are considered core components of a community-based mental health treatment program. Of all the sociodemographic variables analyzed in our study, age was the only parameter related to needs in the presence of MetS. The older participants with MetS identified more total and unmet needs. Meesters et al. [44] found that elderly people with schizophrenia reported similar results and they hypothesized that it is probable that changes associated with aging generate new care needs and/or modify existing ones. Looking at the data in detail, we found the higher number of met needs in the environmental and physical domains. We believe that the association between age and perceived needs in our sample can be explained by the effects of a history of cardiovascular disease, diabetes and dyslipidemia. The association between needs and sociodemographic variables is inconsistent [10, 13, 45].

In our study, we found scores and needs domains similar to those of other studies [6, 7, 10–12, 45, 46]. Arvidsson [10] found that both staff and patients rated the most common needs in the domains related to psychotic symptoms, psychological distress, looking after the home and company. However, the patients rated a mean score of total needs that was lower than ours. McCrone at al. [11] provided further comparisons of needs in patients with schizophrenia in 5 different European countries. Despite their specificities, the between-site differences in overall met needs were very small, while unmet needs varied substantially. The majority of the participants from Santander (Spain) were living with their relatives and accommodation was thus not a problem; daytime activities, however, were more frequently reported as a problem than in the other cities. One of the underlying causes may have been the lack of alternative structures for day care and psychosocial rehabilitation. We found that drugs and alcohol in the CAN interview domains are rarely mentioned as areas of need due to a possible underreporting of data by the participants. A description of the most frequently identified unmet needs can be found in the recent review of Torres-González et al. [25].

In our study, the individuals with MetS identified more perceived needs compared to the group without this condition. However, we found no correlation between the number of needs and the presence of MetS, probably due to the strict control of physical health problems by general practitioners who treat the patients jointly with mental health professionals. We believe that this comprehensive approach minimizes the possible effect of MetS in the perception of needs for care expressed by the participants.

HRQoL did not show a significant association with MetS. However, mobility problems were negatively associated with the number of total, met and unmet needs. While EQ-5D is a generic HRQoL instrument that explores the perception of health on the day of the interview, CAN explores needs over the previous month and this may be a reason for this unexpected result. We came to the conclusion that EQ-5D is probably not suitable for this kind of study because the influence of physical and/or mental problems and acute and/or chronic needs on responses is unclear.

HRQoL was related to the number of total and unmet needs in participants with MetS. A strong relationship exists between unmet needs and QoL [13, 14, 45, 47, 48]. Hansson and colleagues [15] found that unmet needs were associated with poorer QoL and Lambri et al. [16] found that “the observer-rated psychiatric symptom score did not determine QoL, although self-reported psychopathology and social need did”. Meesters et al. [44] found that unmet needs were associated with poorer QoL in elderly patients with schizophrenia. Although some studies have shown an association between schizophrenia and physical problems (including MetS) and QoL [21, 30, 49, 50], Medeiros-Ferreira et al. [23] found no correlation between MetS and HRQoL. They did, however, find that people who engaged in physical activity reported better self-perceived health and this can be understood as a feature of HRQoL.

We found that global functioning was significantly related to needs in participants without MetS. Positive symptomatology was related to the number of total and met needs in people with MetS and general symptomatology was associated with the number of total needs. This finding might be attributable to the fact that our study sample consisted of outpatients whose needs were satisfied by the health care interventions provided with respect to dealing with positive symptoms but not with general symptoms (such as anxiety or depression) associated with perceived needs. Ochoa et al. [6] and Alvarado et al. [45] found similar results. However, it was not the aim of these studies to analyze the presence of MetS and its association with needs and HRQoL.

Our findings should be interpreted with caution. This was a cross-sectional study and the fact of the possible overlapping definitions of needs and HRQoL, make it more difficult to address the differences between the influence of one or another on the results. Nevertheless, the association of a higher number of unmet needs with a lower perception of QoL has emerged as a robust finding in other studies [16, 44, 48, 50]. Further, it has not been possible to analyze the sample with schizophrenia or schizoaffective disorder separately, the same has occurred with the stratification by typical or atypical antipsychotics due to the limited number of patients enrolled in the study. As our sample was composed of outpatients receiving treatment in a public mental health center, the results cannot necessarily be generalized to people with other profiles.

The main strength of our study lies in the fact that it was based on “real-world” clinical practice and exclusively on the participants’ own perceptions of needs. It is well known that the assessment of needs by patients, staff and caregivers provides a more comprehensive evaluation and that mental health interventions must be chosen based on a negotiation process between them [10, 44, 51]. Although we totally agree with this perspective, we believe that assessing only the perceived needs and HRQoL of individuals suffering from schizophrenia or schizoaffective disorder is a way of empowering them in their own decision-making process in dealing with the illness. Indeed, in public health studies the patient’s perspective sheds light on how people deal with a chronic disease and is given the same importance as the professional perspective. In this respect, we disagree with the administration of the CAN interview only to staff, which is less time-consuming, but may be not sufficient and appropriate for clinical routine.

## Conclusions

To the best of our knowledge, our study was the first to analyze the relationship between perceived needs, HRQoL and MetS in patients with schizophrenia or schizoaffective disorder. This has implications for treatment planning at the individual and organizational levels. The future plan is to develop individualized lifestyle programs for individuals with this profile based on their specific needs, for example, changing the control of positive symptomatology as main focus to a more open perspective that includes interventions in general symptomatology (e.g. somatic concern, anxiety or poor attention). An analysis of both physical and mental needs could provide a starting point for the extension of facilities in the health care system in order to reach the goal of improving QoL.

## Abbreviations

BMI: Body Mass Index
CAN: Camberwell assessment of need
CATIE: Study- clinical antipsychotic trials intervention of effectiveness study
EQ-5D: EuroQol-5 Dimensions
GAF Scale: Global Assessment of Functioning Scale
HRQoL: Health-Related Quality of Life
MetS: Metabolic syndrome
NCEP-ATP-III criteria: National cholesterol education program- third adult treatment panel- criteria
PANSS Scale: Positive and negative syndrome scale, divided into positive (PANSS-P), negative (N) and general (G) symptomatology
QoL: Quality of life
SCID-I: Structured clinical Interview for the DSM-IV-Axis I disorders
